# Reference Ranges for Hemoglobin and Hematocrit Levels in Neonates as a Function of Gestational Age (22–42 Weeks) and Postnatal Age (0–29 Days): Mathematical Modeling

**DOI:** 10.3390/children6030038

**Published:** 2019-03-01

**Authors:** Felix Scholkmann, Daniel Ostojic, Helene Isler, Dirk Bassler, Martin Wolf, Tanja Karen

**Affiliations:** 1 Biomedical Optics Research Laboratory, Department of Neonatology, University Hospital Zurich, University of Zurich, 8091 Zurich, Switzerland; Daniel.Ostojic@usz.ch (D.O.); Helene.Isler@usz.ch (H.I.); Martin.Wolf@usz.ch (M.W.); 2 Department of Neonatology, University Hospital Zurich, University of Zurich, 8091 Zurich, Switzerland; Dirk.Bassler@usz.ch (D.B.); Tanja.Karen@usz.ch (T.K.)

**Keywords:** hematology, hemoglobin, hematocrit, reference intervals

## Abstract

Hematological values of neonates need to be interpreted taking into account the fact that the reference ranges depend on the age of the neonate. We aimed to derive two general mathematical models for reference ranges for hemoglobin concentration (cHb) and hematocrit (Hct) levels in neonates as a function of gestational age (GA) and postnatal age (PNA), since it is known that GA and PNA are independent factors determining cHb and Hct. For this purpose, cHb and Hct values from the data set of Henry and Christensen (2015, *Clin. Perinatol*., 42, 483–497) from about 100,000 neonates (GA: 22–42 weeks, PNA: 0–28 days) were used and general models with two quadratic functions were derived. To the best of our knowledge, the models we have developed are the first published ones to provide reference ranges for cHb and Hct for neonates incorporating the parallel dependence on GA and PNA.

## 1. Introduction

The iron-containing protein hemoglobin (Hb) plays a fundamental part in human physiology, enabling oxygen transport and having non-oxygen-related functions [1]. Both the Hb concentration (cHb) as well as the volume of red blood cells compared to the total blood volume, i.e., the hematocrit (Hct), are important hematologic parameters for medical diagnosis.

For adult humans, normal ranges for cHb and Hct are established. For neonates, however, the availability of normal ranges is limited. This is due to ethical concerns of drawing blood from neonates purely for research purposes, which hinders the collection and measurement of samples to obtain normal values [2,3,4,5,6,7,8]. Therefore, surrogates of normal ranges, i.e., *reference ranges*, were defined and published based on measurement of clinically stable neonates.

**Figure 1:**
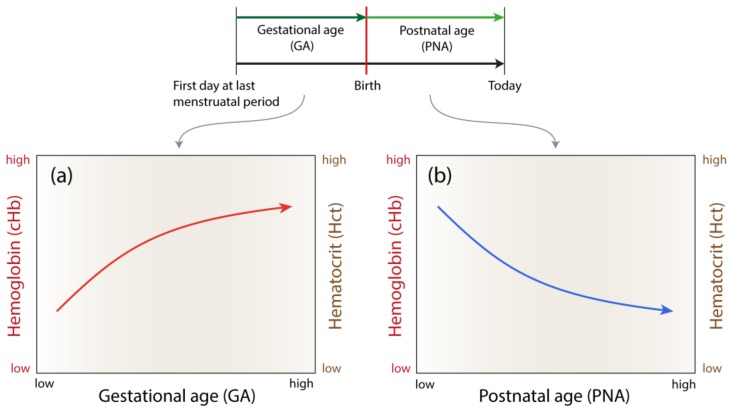
cHb and Hct trend in neonates depending on gestational age (**a**) and postnatal age (**b**) according to the findings of Henry and Christensen [6].

Knowledge of cHb and Hct reference ranges for neonates (term born or preterm born) is important to evaluate the normal development and to detect abnormal physiological conditions, e.g., anemia which might require a red blood cell transfusion [9,10]. The reference ranges provided from the largest (approximately 100,000 neonates) and most up-to-date study by Henry and Christensen in 2015 [6], showed clearly that cHb and Hct depend on the age of the neonates in a specific manner: an *increase* of cHb and Hct with gestational age (GA) and a *decrease* with postnatal age (PNA, i.e., days of life) (see Figure 1). This highlights the fact that GA and PNA are *independent* factors that determine the cHb and Hct values in neonates. Thus, for a comparison of measured cHb and Hct values with reference ranges, both the GA and PNA need to be considered. Surprisingly, this is hardly done in clinical practice, and studies generally consider only one of these factors. According to our understanding, one main reason for this is that there is no published model describing the cHb and Hct reference ranges as a function of both GA and PNA.

Our aim was to derive such general models of cHb and Hct reference ranges depending on GA and PNA, based on the data published by Henry and Christensen.

## 2. Materials and Methods

### 2.1. Hemoglobin and Hematocrit Data

The mathematical models were derived by extracting cHb and Hct data from Figures 1–4 of [6] using the WebPlotDigitizer software. The data comprised mean and percentile values (5th and 95th) of cHb and Hct depending on GA (22–42 weeks) and PNA (0–28 days) as shown in Figures 1–4 of Henry et al. [6]. The data set only contained values from neonates who had no chromosomal abnormality (based on ICD9; International Classification of Disease, 9th Revision) and had not received a red blood cell transfusion prior to the blood tests. According to Henry and Christensen, the data sets were based on the measurement of about 100,000 neonates and more than 350,000 individual test values [6]. The data was collected between 2005 and 2014 at the Intermountain Healthcare system, USA. The final data sets created were: cHb as a function of GA and PNA, and Hct as a function of GA and PNA. The data sets contained the mean and percentile values. The way we extracted and processed the data was approved by Prof. R.D. Christensen (University of Utah, USA).

### 2.2. Derivation and Validation of the General Models

A genetic programming-based symbolic regression (GP-SR) tool (Eureqa [11,12]) was used first to determine the best fitting function for the two data sets. A bi-quadratic fit with 5 free parameters was found to be the optimum compromise between accuracy and complexity of the models. The final fitting of the functions was then performed in Matlab (The Mathworks, Natick, MA, USA) by nonlinear least-squares fitting with Levenberg-Marquardt minimization.

The models’ quality were evaluated by calculating the mean absolute error (MAE) between the predicted mean cHb and Hct values based on equations 1 and 2 and (i) the data of Henry and Christensen [6], and (ii) cHb and Hct values of clinically stable preterm neonates without congenital malformations (*n* = 19, GA: 33.91 ± 1.73, PNA: 8.26 ± 7.31; capillary blood samples) measured in our department as part of a neuroimaging study (approved by the Ethical Committee of Zurich, KEK 2010-0102/2, and Swissmedic, 2010-MD-0019).

## 3. Results

### 3.1. The General Models

The two models derived for the mean obtained reference values of cHb $\left({\mathrm{cHb}}_{\mathrm{Ref}}^{\mathrm{Mean}}\right)$ and Hct $\left({\mathrm{Hct}}_{\mathrm{Ref}}^{\mathrm{Mean}}\right)$ are:

(1) $${\mathrm{cHb}}_{\mathrm{Ref}}^{\mathrm{Mean}}=\alpha_{1}+\alpha_{2}\;\mathrm{GA}+\alpha_{1}\;\mathrm{PNA}+\alpha_{3}\;{\mathrm{GA}}^{2}+\alpha_{4}\mathrm{GA}\;\mathrm{PNA}+\alpha_{5}\;{\mathrm{PNA}}^{2},$$

(2) $${\mathrm{Hct}}_{\mathrm{Ref}}^{\mathrm{Mean}}=\beta_{1}+\beta_{2}\;\mathrm{GA}+\beta_{1}\;\mathrm{PNA}+\beta_{3}\;{\mathrm{GA}}^{2}+\beta_{4}\mathrm{GA}\;\mathrm{PNA}+\beta_{5}\;{\mathrm{PNA}}^{2},$$

with *α* and *β* free parameters given in Table 1.

**Figure 2:**
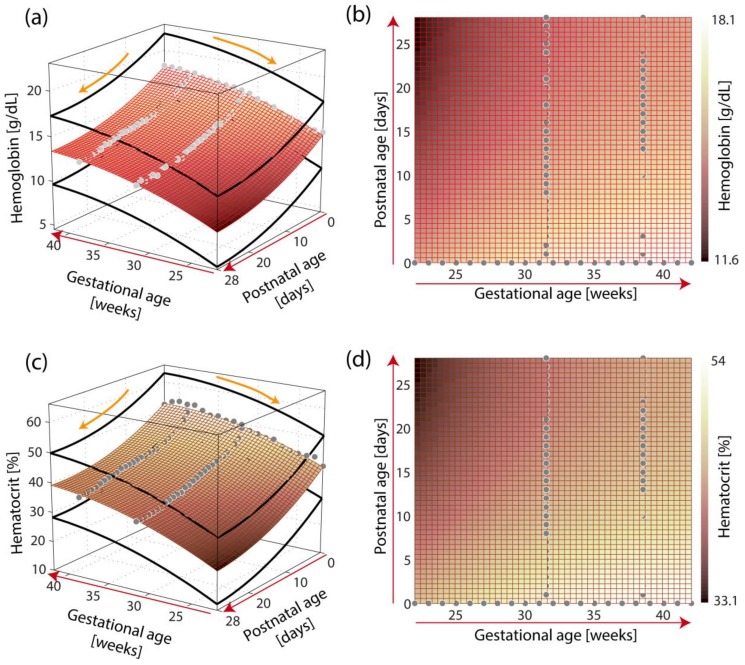
Visualization of the models of the reference ranges of hemoglobin concentration (cHb) (**a**,**b**) and hematocrit (Hct) (**c**,**d**) as a function of gestational age (GA) and postnatal age (PNA). The models are visualized in 3D (a,c) and 2D (**b**,**d**). The empirical data (cHb: *n* = 77, Hct: *n* = 77) of Henry and Christensen [6] is shown as gray dots. The thick black lines in (**a**) and (**c**) represent the 5–95th percentile range.

If was found that the percentile function is modeled by a constant factor added to equations (1) and (2) due to the constant symmetry of the value distributions with respect to GA and PNA:

(3) $${\mathrm{cHb}}_{\mathrm{Ref}}^{5\mathrm{th}}={\mathrm{Hb}}_{\mathrm{Ref}}^{\mathrm{Mean}}-\gamma$$

(4) $${\mathrm{Hb}}_{\mathrm{Ref}}^{95\mathrm{th}}={\mathrm{Hb}}_{\mathrm{Ref}}^{\mathrm{Mean}}+\gamma$$

(5) $${\mathrm{Hct}}_{\mathrm{Ref}}^{5\mathrm{th}}={\mathrm{Hct}}_{\mathrm{Ref}}^{\mathrm{Mean}}-\lambda$$

(6) $${\mathrm{Hct}}_{\mathrm{Ref}}^{95\mathrm{th}}={\mathrm{Hct}}_{\mathrm{Ref}}^{\mathrm{Mean}}+\lambda$$

with *γ* = 3.76 and *λ* = 10.69. Figure 2 shows a visualization of the models.

### 3.2. Validation of the General Models

The comparison of the predicted cHb and Hct values based on equations 1–6 with the data of Henry and Christensen showed a small MAE of 0.162477 g/dL for cHb and 0.683472% for Hct. The results support the goodness-of-fit of the derived models with the source data used to construct the models.

**Figure 3:**
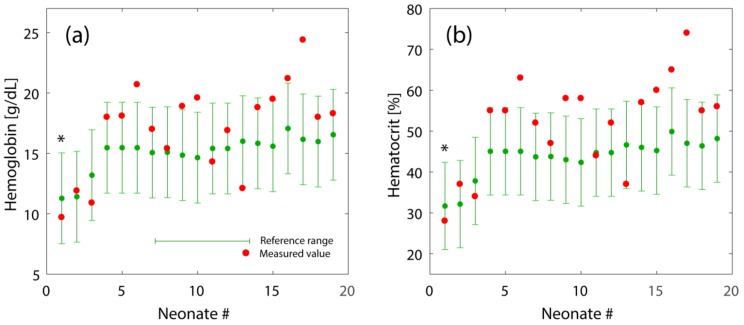
Reference range of cHb (**a**) and Hct (**b**) obtained by equations 1–6 along with measured cHb and Hct values from clinically stable preterm neonates (*n* = 19) of different ages (GA and PNA). The order of neonates is shown with increasing GA. The asterisk indicates one neonate that received a blood transfusion.

The validation with the second data set (i.e., cHb and Hct data from 19 preterm neonates) showed that 63.2% of the cHb and Hct values where within the predicted 5–95th percentile ranges (see Figure 3).

## 4. Discussion, Conclusion and Outlook

We report the derivation of general models for cHb and Hct reference ranges depending on GA (22–42 weeks) and PNA (0–29 days). The models were validated using the source data from Henry and Christensen as well as a data set of cHb and Hct values of 19 preterm infants included in a study of our own measuring cerebral oxygenation [13]. The comparison of our own measured cHb and Hct values to the reference ranges showed that there were several values above the reference ranges. One explanation might be that the cHb and Htc values published by Henry and Christensen represent a mixture of different sources of blood samples (capillary, venous, arterial) [5], whereas our blood samples were solely capillary. Daae at al. [14] reported cHb and Htc values of capillary blood to be 2% higher than from venous blood, where Yang at al. [15] found no significant difference in cHb and Hct values between venous and capillary blood in contrast to arterial versus venous blood parameters (a 3.1% higher Hct in venous blood compared to arterial blood). The method of analysis (venous vs. capillary blood) seems to have no significant effect on these two values [16,17]. We therefore conclude that our models are still clinically useful even in the case when cHb and Hct values from different sources (arterial, venous, capillary) are used. The fact that some of the own measured cHb and Hct values were not in the reference ranges might be due a insufficiency of our model but also could indicate an increased individual variability of the physiology of some newborns which is detected when comparing to a reference range based on data of a large population sample (as in our case with data from about 100,000 neonates).

Our models provide reference ranges for cHb and Hct for neonates incorporating the parallel dependence on GA and PNA. Previously reported studies showed clearly that GA and PNA are independent factors determining the cHb and Hct values in neonates [6].

The main problem for preterm and term born infants is the limited availability of reference ranges [18]. Obladen et al. [19] tried to characterize a hematologic profile for very low birth weight infants (VLBW < 1500 g). For day 3 of life, cHb was found to be 15.6 (11.0, 19.8) g/dL ( median, 3rd percentile, 97th percentile) and Hct to be 47 (35,60). With increasing age, the values declined. The limitation of this study was that all VLBW infants received one or more red blood cell transfusions after day 3 of life.

All neonates experience a decline in circulating red blood cells during the first weeks of life, but physiological changes in cHb and Hct concentration during the neonatal period of preterm infants are difficult to interpret because of the confounding issues of periodic phlebotomy losses and blood transfusions [20,21,22]. When the results of a neonate´s complete blood count (CBC) is examined, clinicians sometimes struggle to identify precisely which elements are normal, and which are abnormally low or high. Making a judgement about whether a hematological value (such as hematocrit) is normal, low or high frequently depends on the neonate´s GA at birth and on the PNA when the blood was drawn.

The fact that cHb is decreasing with PNA might, to a specific degree, also be due to the fact that the drawing of blood changes the cHb of the neonate. Depending on the total blood volume used for the analysis and the time at which blood was drawn, the cHb will change, which could also explain the variability between study results.

The models developed in this paper with the predicted reference ranges for cHb and Hct can be used in clinical practice to compare actually measured cHb and Hct values with the reference ranges in order to ascertain possible deviations from the reference. It helps the clinicians to obtain confidence that each CBC parameter is being interpreted properly.

One limitation of the reports on reference ranges of cHb and Hct in the neonatal period is the fact that preterm infants <29 weeks of gestation and with anemia and those who received a blood transfusion were excluded [20,21,22]. As infants with extremely low birth weight uniformly develop anemia of prematurity and frequently require multiple red blood cell transfusions during neonatal intensive care [9,10] it might be of additional value to provide reliable neonatal reference ranges to inform the decision of when to perform a transfusion on a preterm infant. Also useful would be reference ranges for post-transfusion, considering the life span of erythrocytes transfused to preterm infants [23]. Besides the comparison with reference ranges, however, knowledge of correct transfusion thresholds is of particular significance. A large study (ETTNO study) investigating the effects of transfusion threshold on neurocognitive outcome is currently being conducted [24].

Furthermore, an interesting extension of the models would be to increase the time intervals for which the models are valid and to incorporate also birth weight, while subcategorizing to a small appropriate size for gestational age (including all low birth weight infants), which is also a factor determining cHb values in neonates [18,25]. Also distinguishing between fetal and adult hemoglobin as well as the measurement of immature platelet fraction parameters would be helpful to better model the postnatal hematological changes. With the increasing use of novel automated hematology analyzers, these data will be available in the future, as already shown in a recently published study [26].

Future projects that provide neonatal CBC reference ranges in real time, embedded in smart programs to aid diagnosis and treatment, could reduce clinical variability, reduce costs of care, and improve outcomes. The models presented here can assist clinicians who seek to determine whether values obtained on their neonatal patients fall within or outside the reference ranges.

An implementation of the models in Excel, Matlab and R is freely available on the website of our institution (http://www.neonatologie.usz.ch).

## Figures and Tables

**Table 1:** Parameter values for equations (Eq.) 1 and 2, relating ${\mathrm{cHb}}_{\mathrm{Ref}}^{\mathrm{Mean}}$ and ${\mathrm{Hct}}_{\mathrm{Ref}}^{\mathrm{Mean}}$ with GA and PNA.

Variable	Eq. No.	Parameters				
${\mathrm{cHb}}_{\mathrm{Ref}}^{\mathrm{Mean}}$	1	*α*_1_ = −0.3409	*α*_2_ = 0.8300	*α*_3_ = −0.0093	*α*_4_ = 0.0003	*α*_5_ = 0.0058
${\mathrm{Hct}}_{\mathrm{Ref}}^{\mathrm{Mean}}$	2	*β*_1_ = −1.2956	*β*_2_ = 2.5369	*β*_3_ = −0.0300	*β*_4_ = 0.0069	*β*_5_ = 0.0190
